# Use of reduced dose rate when treating moving tumors using dynamic IMRT

**DOI:** 10.1120/jacmp.v12i1.3276

**Published:** 2010-09-20

**Authors:** Laurence Court, Matthew Wagar, Madeleine Bogdanov, Dan Ionascu, Deborah Schofield, Aaron Allan, Ross Berbeco, Tania Lingos

**Affiliations:** ^1^ Department of Radiation Oncology Dana‐Farber/Brigham & Women's Cancer Center Boston MA USA

**Keywords:** interplay effect, organ motion, IMRT

## Abstract

The purpose was to evaluate the effect of dose rate on discrepancies between expected and delivered dose caused by the interplay effect. Fifteen separate dynamic IMRT plans and five hybrid IMRT plans were created for five patients (three IMRT plans and one hybrid IMRT plan per patient). The impact of motion on the delivered dose was evaluated experimentally for each treatment field for different dose rates (200 and 400 MU/min), and for a range of target amplitudes and periods. The maximum dose discrepancy for dynamic IMRT fields was 18.5% and 10.3% for dose rates of 400 and 200 MU/min, respectively. The maximum dose discrepancy was larger than this for hybrid plans, but the results were similar when weighted by the contribution of the IMRT fields. The percentage of fields for which 98% of the target never experienced a 5% or 10% dose discrepancy increased when the dose rate was reduced from 400 MU/min to 200 MU/min. For amplitudes up to 2 cm, reducing the dose rate to 200 MU/min is effective in keeping daily dose discrepancies for each field within 10%.

PACS number: 87.55Qr

## I. INTRODUCTION

Several authors have studied the dose discrepancies caused by the interplay between moving radiation fields (as with dynamic IMRT [sliding window]) and respiratory‐induced motion of the tumor. It has been shown that certain combinations of MLC sequence and target motion can give large dose discrepancies^(^ ^1^ ^,^ ^2^ ^)^ for individual fractions. These effects generally average out when multiple fractions are used,^(^ ^3^ ^–^ ^9^ ^)^ but Seco et al.^(^ ^1^ ^)^ found that this is not always the case (e.g., for step‐and‐shoot IMRT) and, for some situations, a dose error could still remain after multiple fractions.

Previously published work has shown that the daily dose discrepancies caused by the target/MLC motion are dependent on specific characteristics of the MLC and tumor motion.^(^ ^2^ ^,^ ^10^ ^)^ These effects are smallest for large MLC separation, small displacement between adjacent MLCs, slow MLC speeds, short target periods and small target amplitudes. A number of groups have provided specific recommendations on the MLC parameters that will minimize the interplay effect. Yu et al.^(^ ^2^ ^)^ recommends keeping the speed of collimator (or MLC) motion below $0.5\;\text{cm}\;s^{-1}$ for apertures larger than 1 cm. Court et al.^(^ ^10^ ^)^ gives recommendations for the maximum collimator speed as a function of various parameters such as field aperture and target period. For apertures larger than 1 cm the results of Court et al. are similar to those of Yu et al. For smaller apertures (<1 cm), they showed that the collimator speed should be kept to $0.1-0.2\;\text{cm}\;s^{-1}$. It is difficult, however, to use these recommendations to directly guide the planning process. Instead, the following simple guidelines can be used:^(^ ^11^ ^)^

1. Choose gantry and collimator angles that will minimize large fluence gradients (e.g., do not have beams for which portions of the field‐of‐view includes the cord or other critical structures). This helps avoid complicated MLC sequences with small separation between opposing MLCs and large displacement between adjacent MLCs.
2. Avoid pushing the IMRT optimization process too much. This is a somewhat subjective guideline, but it indicates to the planner that the use of unusually high weights (priorities) or low fluence smoothing may result in fields with MLC sequences that have small separations.
3. Use reduced dose rates (to give lower collimator speeds). This has also been suggested by Jiang et al.^(^ ^12^ ^)^

The purpose of this work is to experimentally investigate Guideline #3. Specifically, we investigated the impact of reducing the dose rate from 400 MU/min to 200 MU/min. We evaluated the impact of target motion on the daily delivered dose for 20 IMRT lung plans (91 fields) created using these guidelines. Fifteen of these plans are dynamic IMRT plans; five of them are hybrid IMRT plans^(^ ^13^ ^,^ ^14^ ^)^ which use both conformal and IMRT fields to treat the target. Hybrid plans are sometimes preferred to full IMRT plans because they can reduce the volume of lung receiving low doses, and also because they have the potential to reduce the magnitude of dose deviations due to intrafraction motion.^(^ ^14^ ^)^

## II. MATERIALS AND METHODS

### A. Treatment plans

Fifteen separate dynamic IMRT plans and five hybrid IMRT plans were created for five patients (three IMRT plans and 1one hybrid IMRT plan per patient) using Eclipse 8.6 (Varian Medical Systems, Palo Alto, CA). Use of the patients' CT and contour data was approved by an Institutional Review Board. In all cases the daily tumor dose was 200 cGy. Gantry angles for the IMRT plans were either (1) five coplanar, equally spaced angles, (2) 4–6 gantry angles selected to be the same as used in the patient's original conformal plan, or (3) 4–5 angles selected by the planner based on experience and the above guidelines. There were no specific guidelines for collimator rotation. For the hybrid IMRT plans, 50%–60% of the daily dose was delivered using static conformal fields (typically AP:PA), and the remainder was delivered using 3–4 dynamic IMRT fields. The treatment planner created these plans using the above guidelines, but was not given any additional instructions about trying to minimize the effects of motion. Criteria for PTV coverage and critical organ constraints were the same as used in our clinical practice.

### B. 4D IMRT QA

Each field was evaluated using an experimental QA technique which includes the effects of tumor motion on the delivered dose. This technique is fully described and evaluated elsewhere,^(^ ^15^ ^)^ and is summarized here. The field under investigation is delivered using a dose rate of 100 MU/min to a phantom comprising an ion chamber array (MatriXX; IBA Dosimetry America, Bartlett TN), and 3 cm solid water buildup. The ion chamber array comprises a 32×32 grid of ion chambers (volume: $0.08\;{\text{cm}}^{3}$), with center‐to‐center distance of 7.62 mm. It can be configured to take movies (0.2 sec frames) of the delivered dose. Each dose frame is first interpolated to a 1 mm grid. The expected dose distribution is then calculated by summing all the individual dose frames, and blurring this cumulative dose with the respiration trace. The effect of tumor motion on the delivered dose is calculated by shifting each dose frame according to a respiratory motion trace, and the cumulative dose calculated. The delivered dose (with simulated motion) is then compared with the expected dose and the dose error calculated. This process is repeated for 10 evenly‐spaced starting points in the respiration cycle, and finally the fraction of the high‐dose region (defined as the area within the 50% isodose on the expected dose distribution), which always passes a certain criteria (e.g., 5%), is calculated. This calculation is the percentage of pixels for which the daily dose error is always less than the criteria for all starting points in the respiration trace. An important characteristic of this technique is that after the dose movie is taken using a dose rate of 100 MU/min, the effect of different dose rates can be evaluated by changing the time stamp for each movie frame. Note that we use criteria of 5% and 10% because it is unclear what dose error is acceptable on a daily basis for individual fields. The use of these two criteria will allow readers to make a judgment based on their own clinical experience.

Each field was evaluated using the above QA technique used to evaluate the effects of tumor motion with 3 and 5 second periods, and 1.5 and 2.0 cm peak‐to‐peak motion, for dose rates of 200 MU/min and 400 MU/min. Target motions of this size are found in real patients,^(^ ^16^ ^)^ but these are towards the upper range of amplitudes reported in the literature. These values were selected because the QA technique described above has been shown to give good agreement with film for 2 cm peak‐to‐peak motion,^(^ ^15^ ^)^ but is less reliable for 0.5 cm motion, presumably because of the relatively course grid size of the ion chamber array (7.6 mm). We separately repeated the field evaluations for 0.5 and 1.0 cm motions, but the results will be less reliable and are therefore reported qualitatively in the discussion section.

The dose rates were selected to represent the dose rate used for most IMRTs in our clinic (400 MU/min) and a lower rate might be selected if the effects of motion are large. Each field was evaluated separately, with the gantry angle set to zero degrees. In all cases the motion was in the cranio‐caudal direction, and modeled as a ${\text{sin}}^{6}$ function (the motivation for this choice is described in the discussion section). This means that the QA for each field was completed for four different tumor motion combinations. We made the decision to evaluate each field separately rather than evaluate the cumulative dose due to all fields in a given plan because one of the aims of the study is to identify outliers. That is, we are trying to identify situations where there may be patients for which dose discrepancies due to the interplay effect is unacceptably high. Evaluation of individual fields gives us more data to evaluate for these situations. It does, however, give conclusions that are very conservative, as typically the dose discrepancy per fraction can be expected to be less than the dose discrepancy per field.

## III. RESULTS

### A. Treatment plans

Table 1 summarizes the main parameters of the treatment plans. It can be seen that the plans have a large average MLC separation, much larger than the 1 cm minimum suggested in published work to minimize the interplay effect. For the hybrid plans, the IMRT fields provided an average of 42% (range 38%–45%) of the total dose (dose to a reference point in the center of the PTV). The hybrid plans tended to have slightly smaller average MLC separation and lower MUs than the plans which used only IMRT fields (see Table 1). This can be attributed to the fact that the IMRT fields in the hybrid plans are supplying less dose (so lower MUs) and are having to fill in deficiencies in the base conformal plans (giving slightly more complex MLC sequences with smaller MLC separations).

**Table 1:** Summary of the main parameters for the IMRT fields evaluated in this study.

*Parameter*	*Plan Type*	*Mean*	*Standard Deviation*	*Range*
Collimator Angle (degrees)	IMRT	14	24	330 ‐ 60
	Hybrid	0	0	0 ‐ 0
X Field Size (cm)	IMRT	10.9	2.0	7.1 ‐ 14.0
	Hybrid	12.5	1.7	9.8 ‐ 14.8
MU (individual fields)	IMRT	135	38	79 ‐ 251
	Hybrid	89	20	67 ‐ 154
Average MLC Separation (cm)	IMRT	3.4	0.7	1.8 ‐ 4.9
	Hybrid	2.7	0.6	0.8 ‐ 3.5

### B. 4D IMRT QA

The maximum dose errors found in all fields, for different dose rates and different respiratory motions, are shown in Table 2. The maximum error increases with peak‐to‐peak motion and also increases when the target period increases from 3 to 5 sec. The fields from hybrid plans have higher maximum dose errors than the fields from IMRT plans. The maximum dose error was seen to decrease when the dose rate was reduced from 400 MU/min to 200 MU/min.

**Table 2:** Maximum dose errors found for single fields. The data for the hybrid plans is for the IMRT fields only.

*Dose Rate (MU/min)*	*Period (s)*	*Plan Type*	*Peak‐to‐Peak Motion (cm)*	
			*1.5*	*2.0*
400	3	IMRT	12.6%	13.3%
		Hybrid	13.7%	16.2%
400	5	IMRT	15.7%	18.4%
		Hybrid	30.1%	31.8%
200	3	IMRT	3.9%	4.9%
		Hybrid	5.0%	5.2%
200	5	IMRT	9.5%	10.3%
		Hybrid	12.0%	13.5%

The percentage of treatment fields for which more than 98% of the target area (defined as the area within the 50% isodose) never fails 5% or 10% criteria is shown in Table 3 for different dose rates and respiratory motions. Daily dose discrepancies increased in magnitude and frequency, particularly for a target period of 5 sec. In all cases, reducing the dose rate from 400 MU/min to 200 MU/min resulted in all fields passing the 10% criteria, and large increases in the number of fields passing the 5% criteria.

**Table 3:** The percentage of fields for which 98% of the target area (defined using the 50% isodose line) never experienced a dose error larger than the stated criteria (5% or 10%) – that is, the percentage of fields which pass the criteria for all starting points in the respiratory cycle. The data for the hybrid plans is for the IMRT fields only.

*Dose Rate (MU/min)*	*Pass Criteria*	*Period (s)*	*Plan Type*	*Peak‐to‐Peak Motion (cm)*	
				*1.5*	*2.0*
400	5%	3	IMRT	90	88
			Hybrid	0	0
400	5%	5	IMRT	18	5
			Hybrid	0	0
400	10%	3	IMRT	100	100
			Hybrid	92	75
400	10%	5	IMRT	85	76
			Hybrid	33	8
200	5%	3	IMRT	100	96
			Hybrid	100	100
200	5%	5	IMRT	100	93
			Hybrid	42	17
200	10%	3	IMRT	100	100
			Hybrid	100	100
200	10%	5	IMRT	100	100
			Hybrid	100	100

## IV. DISCUSSION

This work has shown that, for the fields evaluated here, the maximum daily dose discrepancy per field when treated with a dose rate of 400 MU/min was 18% for IMRT fields (2 cm peak‐to‐peak motion). The maximum dose discrepancy for IMRT fields which were planned as part of a hybrid plan was 32%. The actual difference between the two treatment types is much smaller than this since, for a hybrid plan, only 38%–45% of the total dose comes from the IMRT fields. This means that a worst‐case scenario of 32% dose discrepancy for each IMRT field would translate into less than 15% when weighted by the IMRT contribution. Reducing the dose rate from 400 MU/min to 200 MU/min reduced the maximum dose discrepancy to 10.3% for IMRT plans and 13.5% for the hybrid plans. Again, the actual difference between the two treatment types is smaller than this.

Considering all fields evaluated here, for 200 MU/min and all target motions, the dose to more than 98% of the target area was always within 10%. There will be some points with daily dose errors per field larger than 5%. For comparison, the reader is reminded that a 5% dose discrepancy is not unusual for some dose calculation algorithms when calculating dose to a lung tumor.^(^ ^17^ ^)^ Similarly, hotspots larger than this (10%–20%) are not unusual.

We used the same techniques to evaluate 0.5 and 1.0 cm peak‐to‐peak motion. The coarseness of the detectors in the ion chamber array and associated relatively poor spatial sampling mean that the results for these smaller motions may be less reliable than the results for the larger motions. We found that for motion of 1 cm or less, the maximum dose discrepancy was less than 10% for all fields, and that 98% of the target area was within 5% for all fields. This was for 400 and 200 MU/min. This implies that even if 200 MU/min is used for peak‐to‐peak motion above 1 cm, there is no need to reduce the dose rate if the motion is 1 cm or less. However, because of the uncertainties in the experimental technique for these smaller amplitudes, this must be considered only a tentative conclusion, and more evaluation is necessary.

Most previous studies into the interplay effect have shown that these effects average out after many fractions.^(^ ^4^ ^,^ ^5^ ^)^ We also found that the average of the doses calculated for each individual starting point in the respiratory phase gave a dose distribution that agreed with the expected dose distribution within 1%. However, Seco et al.^(^ ^1^ ^)^ showed that there are situations where although the average dose discrepancy will be small, the dose discrepancy can still be large for a minority of patients. This may happen when there is not an even distribution in the starting point of the respiratory cycle where the beam is turned on. Similarly, several authors have shown that the dose discrepancy per fraction can be expected to be less than the dose discrepancy for individual fields.^(^ ^5^ ^)^ Again, it can be expected that this depends on the combination of points in the respiratory cycle where the individual beams are turned on. The dose for an individual field for an individual fraction, as evaluated here, can be (very) conservatively considered as the worst‐case scenario. That is, if any single field has a maximum dose discrepancy of X%, then the maximum dose discrepancy for any fraction – or even the entire treatment – will be X%. This is a very conservative approach that does not rely on averaging of different starting points in the respiratory cycle. For most patients, the overall dose discrepancies can be expected to be much smaller than those reported here.

One of the possible advantages of hybrid IMRT plans is that they reduce the potential for dose discrepancies due to the interplay effect. We found, however, that the interplay effect is worse for these fields. This can be attributed to the fact that they are somewhat more complicated fields, with smaller average separation between the MLCs and, because they use lower MUs (as treating lower doses), the MLCs move faster. However, if the results for the hybrid IMRT fields of Table 2 are scaled by the relative contribution of the IMRT fields, then the peak‐to‐peak dose discrepancies of the hybrid fields is very similar to that of the regular IMRT fields. In other words, the impact of the interplay effect for hybrid plans is larger for individual fields, but should be similar for the daily treatment when the majority of the treatment is treated using conformal fields.

Therefore, based on this analysis of 91 individual fields and four different respiratory motions, we propose that a reduced dose rate of 200 MU/min is effective in reducing the interplay effect when treating targets with large (e.g., 2 cm) motion. This conservative strategy can be expected to keep maximum daily dose discrepancies within 10% for all the target volume for all fields, and within 5% for the majority of its volume. For an IMRT plan with 500 MU/min, this would increase the treatment time by 75 sec, so it should not have a large impact on the clinical workflow. This work has also demonstrated the use of a new QA technique to evaluate the impact of target motion on the delivered dose. This could be extended to be used as a routine QA method for IMRT plans. If an ion chamber array is already being used for QA, then there would be no additional measurements necessary. The only change would be that before the delivered dose distribution was evaluated, the dose frames would be shifted to simulate motion.

It should be noted that all plans evaluated here used daily prescription doses of 200 cGy. Increasing the daily dose can be expected to result in slower MLC motion and, therefore, reduced daily dose discrepancies. Although for hypofractionated treatments there is less chance for the daily discrepancies to average out, the reduced daily discrepancies mean that the interplay effect is not likely to give significant cumulative dose discrepancies.

The results presented here modeled the patient motion as ${\text{sin}}^{6}$, with all motion in the superior‐inferior direction. ${\text{Sin}}^{6}$ was selected because previous work showed that the interplay effect is worse for ${\text{sin}}^{6}$ than ${\text{sin}}^{2}$ and ${\text{sin}}^{4}$ motion.^(^ ^1^ ^,^ ^10^ ^)^ The majority of motion in the lung is in the superior‐inferior direction.^(^ ^16^ ^,^ ^18^ ^)^ Real patient motion is, however, not as regular as this. There is also a large patient‐to‐patient variation in the actual shape of the respiratory cycle. This work does not include the effects of these variations.

## V. CONCLUSIONS

Interplay between target motion and MLC motion can result in discrepancies in the daily delivered dose. In particular, these increase with increasing target motion. This work has analyzed the effects of tumor motion on the daily delivered dose for 91 fields (dynamic IMRT and hybrid plans), and shown that for amplitudes up to 2 cm, reducing the dose rate to 200 MU/min is effective in keeping daily dose discrepancies for each treatment field within 10%.

## ACKNOWLEDGEMENTS

This work was partially funded by a Kayes Scholars award.
